# Effects of Direct and Pulse Plating on the Co-Deposition of Sn–Ni/TiO_2_ Composite Coatings

**DOI:** 10.3390/ma17020392

**Published:** 2024-01-12

**Authors:** Eleni Rosolymou, Antonis Karantonis, Evangelia A. Pavlatou

**Affiliations:** 1 Laboratory of General Chemistry, School of Chemical Engineering, National Technical University of Athens, 9, Heroon Polytechniou Str., Zografos Campus, GR-15780 Athens, Greece; 2 Laboratory of Physical Chemistry and Applied Electrochemistry, School of Chemical Engineering, National Technical University of Athens, Zografou, GR-15780 Athens, Greece; antkar@central.ntua.gr

**Keywords:** nano-composite, Sn–Ni coatings, TiO_2_ nanoparticles, direct/pulse electrodeposition, microhardness, wear, photocatalysis, corrosion resistance

## Abstract

Sn–Ni alloy matrix coatings co-deposited with TiO_2_ nanoparticles (Evonik P25) were produced utilizing direct (DC) and pulse electrodeposition (PC) from a tin–nickel chloride-fluoride electrolyte with a loading of TiO_2_ nanoparticles equal to 20 g/L. The structural and morphological characteristics of the resultant composite coatings were correlated with the compositional modifications that occurred within the alloy matrix and expressed via a) TiO_2_ co-deposition rate and b) composition of the matrix; this was due to the application of different current types (DC or PC electrodeposition), and different current density values. The results demonstrated that under DC electrodeposition, the current density exhibited a more significant impact on the composition of the alloy matrix than on the incorporation rate of the TiO_2_ nanoparticles. Additionally, PC electrodeposition favored the incorporation rate of TiO_2_ nanoparticles only when applying a low peak current density (J_p_ = 1 Adm^−2^). All of the composite coatings exhibited the characteristic cauliflower-like structure, and were characterized as nano-crystalline. The composites’ surface roughness demonstrated a significant influence from the TiO_2_ incorporation rate. However, in terms of microhardness, higher co-deposition rates of embedded TiO_2_ nanoparticles within the alloy matrix were associated with decreased microhardness values. The best wear performance was achieved for the composite produced utilizing DC electrodeposition at J = 1 Adm^−2^, which also demonstrated the best photocatalytic behavior under UV irradiation. The corrosion study of the composite coatings revealed that they exhibit passivation, even at elevated anodic potentials.

## 1. Introduction

Tin–nickel electrodeposits have attracted significant attention in both scientific and technological contexts among electrodeposited alloys [1,2,3,4]. These deposits, characterized as intermetallic compounds with substantial proportions of both constituents, stand out from most other alloy deposits. This distinction arises from the fact that typical alloy deposits often contain only trace amounts of a secondary metal that is added in order to slightly modify the properties of the primary metal. What differentiates tin–nickel is the fact that its properties bear little resemblance to those of either tin or nickel [5].

Moreover, in recent years, there has been growing scientific and technological interest in the development of metal matrix composite coatings that incorporate TiO_2_ nanoparticles. These composite coatings offer improved mechanical properties, and exhibit intriguing photocatalytic behavior when exposed to UV light [6,7,8,9,10,11,12]. TiO_2_ is a semiconductor that has been extensively studied, and its physicochemical properties can vary depending on factors such as nanoparticle shape, size, and the predominant crystalline phase present. The energy gap, known as bandgap (E_g_), between the valence and conduction bands of anatase TiO_2_ is approximately 3.2 eV. This characteristic makes anatase TiO_2_ highly sensitive to ultraviolet (UV) irradiation, endowing it with self-cleaning and antibacterial properties [13].

The electrodeposition of Ni–Sn/TiO_2_ coatings holds substantial significance, given that these composites exhibit photocatalytic behavior and could be potentially used as antimicrobial surfaces in everyday items [8,14,15]. Moreover, since Sn–Ni alloys are non-allergic to skin, they could serve as substitutes for electroplated nickel on clothing fasteners [16]. Although sufficient reports on the electrodeposition of Sn–Ni coatings already exist [17,18,19], there are relatively few reports focusing on the systematic effects of direct and pulse current electrodeposition on the microstructure, crystal structure, mechanical attributes, and corrosion behavior of Ni–Sn matrix composite coatings [8,15,20]. Various studies have indicated that the utilization of pulse current, as opposed to conventional direct current electrodeposition, enhances particles’ incorporation rates, and improves the particles’ distribution within the metal matrix [21,22].

In this study, the electro-deposition parameters—direct and pulse current—were modified to produce Ni–Sn/TiO_2_ composite coatings. The TiO_2_ nanoparticles utilized were commercial TiO_2_-P25 nanoparticles, known for their efficient photocatalytic performance under UV irradiation. The influence of direct and pulse current density was investigated for two key aspects: (i) the co-deposition rate of TiO_2_ nanoparticles in the matrix and (ii) the composition of the composite coatings’ alloy matrix. The produced Ni–Sn/TiO_2_ composite coatings were also examined in terms of their surface morphology, crystal structure, microhardness, wear and photocatalytic properties, as well as corrosion resistance.

The primary objective of the present study was to optimize the incorporation percentage of TiO_2_ nanoparticles utilizing direct and pulse current electrodeposition. This optimization aimed to produce Ni–Sn/TiO_2_ composite coatings with enhanced mechanical, tribological, photocatalytic, and anticorrosion properties.

## 2. Materials and Methods

### 2.1. Electrodeposition Experiments

Sn–Ni/TiO_2_ coatings were electrodeposited using both direct current (DC) and pulse current (PC) techniques. The electrodeposition process was conducted using the commercial electrolyte Galvaloy NS11, provided by Elplatek A/S (Espergærde, Denmark), which is an aqueous chloride fluoride-based tin–nickel solution [8]. The electrodeposition conditions are outlined in Table 1.

During the electrodeposition experiments, a three-electrode setup was employed. The cathode material consisted of brass discs that underwent mechanical treatment prior to each experiment, while the anode was a soluble nickel plate of 99.9% purity. The reference electrode was a 3M KCl-saturated Ag/AgCl electrode.

As outlined in our previous study [8], when electrodepositing pure tin–nickel coatings with a composition of 65 wt.% Sn–35 wt.% Ni, the bath’s Sn content was kept within the range of 20–25 g/L, while the Ni content fell within the range of 55–65 g/L. To maintain these concentrations during the electrodeposition of the Sn–Ni/TiO_2_ composite coatings, continuous monitoring of both the tin and nickel content was carried out through titration. Following each titration, precise amounts of tin chloride and nickel chloride were added to the solution, in order to maintain the desired levels of tin and nickel.

Additionally, the TiO_2_ nanoparticles’ loading within the electrolytic bath was kept constant at 20 g/L using Evonik P25 TiO_2_ (Evonik Industries AG, Essen, Germany), without any extra dispersant additives required. To ensure that the nanoparticles remained suspended uniformly, magnetic stirring was applied for at least 24 h both before and during the electrodeposition process. To assure consistency, the electroplating bath’s pH was adjusted to constant values within the range of 4.2–4.4 before commencing each electrodeposition experiment.

During direct current electrodeposition, the current density varied within the range of 1 to 5 A · dm^−2^. When employing the pulse current technique, the peak current density (J_p_), also ranged from 1 to 5 A · dm^−2^.

In addition to these parameters, two new parameters were introduced, (a) the duty cycle (d.c.) and (b) pulse frequency (ν). The duty cycle remained constant at 50% and was calculated as T_on_/(T_on_ + T_off_), where T_on_ represents the duration of the applied current pulses and T_off_ corresponds to the relaxation time between pulses. The pulse frequency (ν) varied from 0.1 to 100 Hz.

All electrodeposition experiments were conducted using the potentiostat/galvanostat Autolab PGSTAT302N (Metrohm AG, Herisau, Switzerland). The duration of these experiments was properly adjusted, in order to maintain the thickness of the deposits equal to approximately ~15 μm. To ensure reliability and consistency, the electrodeposition experiments were repeated thrice, and the resulting composite coatings were subjected to thorough examination and analysis.

**Figure 1:**
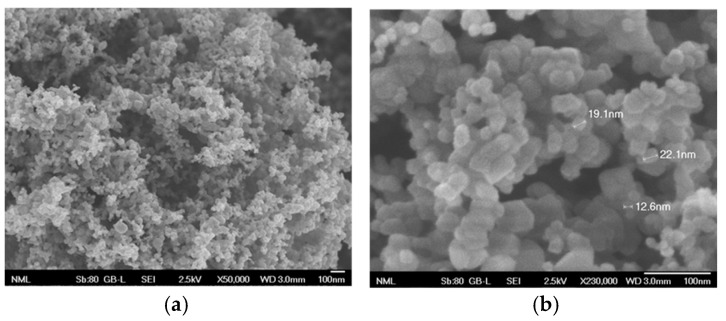
FE-SEM micrographs, at (**a**) magnification (×50,000) and (**b**) magnification (×230,000), of the commercial Evonik P25 TiO_2_ nanoparticles (d = 21 nm).

The Sn–Ni/TiO_2_ composite coatings were fabricated by immobilizing the commercially available Evonik P25 TiO_2_ nanoparticles within the metal matrix. Figure 1a,b illustrate the typical morphology of the Evonik P25 TiO_2_ nanoparticles. These TiO_2_ nanoparticles exhibit a nanocrystalline structure, and possess an average particle size in the nanoscale range.

### 2.2. Structural and Morphological Analysis

The morphological study of the composite coatings was conducted through scanning electron microscopy (FEI QUANTA 200) and high-resolution field emission SEM (JSM-7001F, JEOL, Tokyo, Japan). The alloy matrix composition and TiO_2_ nanoparticles’ concentration incorporated within the alloy matrix were examined using energy-dispersive X-ray spectroscopy (EDS) measurements with a FEI QUANTA 200 (Thermo Fischer Scientific, Hillsboro, OR, USA).

The structural characteristics of the deposits were analyzed using a Siemens (Munich, Germany) D-5000 X-ray diffractometer with a CuKα radiation. Diffractograms were recorded with a step size of 0.2° for 2θ ranging between 20° and 80°. The grain size of the crystallites was determined using the (102) X-ray diffraction peak broadening based on the Sherrer’s equation. The lattice constant of selected composite coatings was evaluated through X-ray diffraction analysis.

The composite coatings’ roughness was measured using a surface roughness tester (Homel Tester T1000, Hommel Werke GmbH, Villingen-Schwenningen, Germany).

### 2.3. Photocatalytic Measurements

To assess photocatalytic activity, UV light irradiation was employed. UV light was generated by four parallel Sylvania 15 W blacklight lamps (peak intensity at 368 nm, 830 lumens). Methyl orange (MO) was used as the model pollutant for the photocatalytic degradation experiments under UV light irradiation, which exhibits maximum absorbance at 464 nm. The equipment and test protocol applied were the same as in our previous study, and are thoroughly described in references [8,9].

### 2.4. Mechanical and Wear Resistance Properties

Vickers microhardness measurements (HV in GPa) were carried out using a Reichert microhardness tester under a 25 g load for 15 s, with the final values determined as the average of 10 measurements.

Tribological performance of the composites was studied using ball-on-disc measurements using a CSM tribometer (CSM Instruments, Portland, OR, USA) under dry sliding conditions. The friction coefficient was automatically recorded. The applied tribological experimental conditions are summarized in Table 2. The wear tracks were examined using electron microscopy (JSM-6390, JEOL, Tokyo, Japan) and a laser-type optical profilometer (3D profilometer, Bruker, Billerica, MA, USA). The volumetric wear factor (c_w_) was calculated using the following equation:

c_w_ = V/FS (cm^3^/Nm) (1)

where V is the volume loss determined by the profilometer, F is the applied load, and S is the total sliding distance.

### 2.5. Corrosion Tests

Electrochemical measurements and assessments of the composite coatings’ corrosion behavior were carried out in a 3.5 wt.% NaCl solution using a conventional three-electrode corrosion flat cell, employing a Princeton Applied PAR 62A (Princeton Applied ResearchA, Oak Ridge, TN, USA) corrosion test system. In this setup, the electroplated sample served as the working electrode, a platinum plate was utilized as the auxiliary electrode, and a 3M KCl-saturated Ag/AgCl electrode was used as the reference electrode. The sequence of measurements followed a standardized protocol in all corrosion tests, as follows:

1. Before each measurement, the sample was immersed in the 3.5 wt.% NaCl solution for ≈30 min to stabilize the open circuit potential.
2. LPR (linear polarization resistance) measurement: The polarization resistance (R_p_) was calculated from traces of the polarization curve at ±20 mV from the OCP (scan rate of 0.1660 mV/s), from which the R_p_ slope was estimated after linear fitting. The R_p_ represents the degree of the passivation layer’s protection of the alloy surface.
3. Tafel polarization curves were recorded at least 200 mV below the OCP to 1.2 V vs. Ag/AgCl, with a potential scan rate of 1 mV/s.

## 3. Results and Discussion

### 3.1. TiO_2_ Nanoparticles’ Incorporation into Sn–Ni Matrix

#### 3.1.1. Direct Current Conditions

In our previous study [8], we extensively investigated the impact of doped TiO_2_ nanoparticles’ loading into the electrolyte, and its relationship with the co-deposition rate within the Sn–Ni/TiO_2_ composite coatings when applying direct current electrodeposition. Specifically, we found that increasing the bath loading from 20 g/L to 30 g/L had a negligible effect on the nanoparticles’ incorporation rate into the metal matrix, especially when the mean current density values were in the range of 2–3 A/dm^2^.

**Figure 2:**
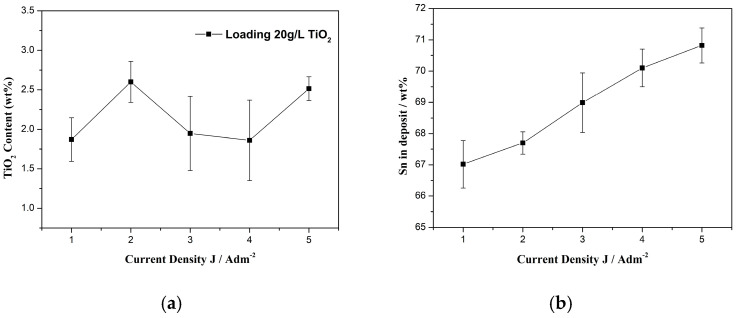
(**a**) Co-deposition rates (wt.%) of TiO_2_ nanoparticles embedded in the tin–nickel matrix and (**b**) Sn content (wt.%) in the Sn–Ni/TiO_2_ composite coating, as a function of the applied current density.

Therefore, in the current study, we maintained a constant loading of 20 g/L of the commercial (non-doped) TiO_2_ Evonik P25 nanoparticles in the electrolyte. As a result, our focus shifted towards examining the impact of varying the applied current density on two key aspects: (i) the co-deposition rate of titania nanoparticles, and (ii) the composition of the alloy matrix within the composite coatings. The impact of the applied current density on the weight co-deposition rate of the TiO_2_ nanoparticles and on the tin content is presented in Figure 2.

The impact of the applied current density, ranging from 1 to 5 A/dm^2^, on the weight co-deposition rate of the TiO_2_ nanoparticles is presented in Figure 2a. As depicted in Figure 2a, the incorporation of TiO_2_ nanoparticles exhibits a distinct pattern. It reaches a maximum in the current density range of 1–3 A/dm^2^, with the highest co-deposition occurring at 2 A/dm^2^. The co-deposition percentage remains relatively constant in the 3–4 A/dm^2^ range, and then starts to increase again at 5 A/dm^2^. Notably, the highest co-deposition percentage, approximately ~2.6 wt.% TiO_2_, was attained at both 2 A/dm^2^ and 5 A/dm^2^.

This behavior of particle incorporation with varying current density aligns with a similar trend observed in the case of alumina particles electrodeposition in a copper matrix [23]. The changes in particle incorporation with current density can be categorized into various distinct regions, each possessing unique characteristics and trends.

Furthermore, the applied current density not only influences the co-deposition rate of nanoparticles, but also has a significant impact on the composition of the metal matrix, as illustrated in Figure 2b. Previous research on electrodeposited Sn–Ni and Sn–Ni/TiO_2_ coatings has indicated that the application of higher current density results in an increase in the tin content (relative to nickel) when the concentration of nickel chloride in the chloride–fluoride bath falls within the range of 60–150 g/L [8,24]. This variation arises from the distinct rate constants associated with the electrode reactions of Sn and Ni. As the cathodic current density increases from 1 to 5 A/dm^2^, Sn reduction occurs at a faster rate at the cathode compared to Ni [25]. However, it is worth mentioning that this change in current density from 1 to 5 A/dm^2^ does not greatly alter the atomic equilibrium ratio of Ni:Sn, which remains approximately 1:1 [4,24].

Remarkably, it was observed that the applications of both the 2 and 5 A/dm^2^ current densities results in a nearly identical nanoparticles’ co-deposition rate within the matrix, while the Sn content increases from 67.5 to 70.5 wt.% (Figure 2b); this is consistent with the findings from our previous study [8]. Overall, these results suggest that the effect of current density plays a more pivotal role in determining the composition of the alloy matrix compared to its influence on the incorporation rate of TiO_2_ in the composite coatings.

#### 3.1.2. Pulse Current Conditions

**Figure 3:**
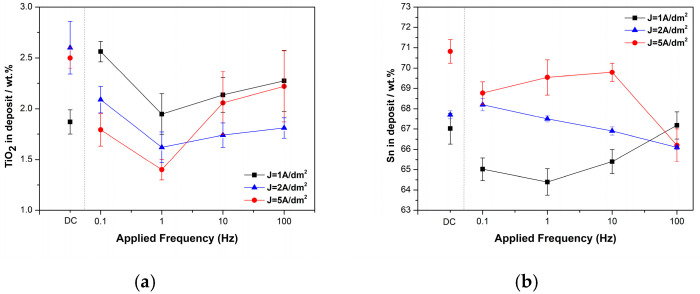
Effect of pulse frequency on the (**a**) co-deposited TiO_2_ nanoparticles (wt.%) and (**b**) Sn content (wt.%) in the deposit.

Pulse electrodeposition was employed with a constant nanoparticles’ loading in the bath (20 g/L) and a fixed-pulse duty cycle equal to 50%. Investigation of the pulse parameters focused on two aspects: (i) varying the pulse current frequency (ν = 0.1 to 100 Hz) and (ii) adjusting the peak current density, J_p_, to 1, 2, and 5 A/dm^2^. The selection of the applied pulse current densities was based on the minimum and maximum TiO_2_ co-deposition percentage achieved under DC conditions. Figure 3a,b illustrate the weight percentage (wt.%) of the incorporated TiO_2_ nanoparticles and the Sn content within the Sn–Ni matrix as a function of the pulse frequency at various peak current density values.

The data presented in Figure 3a indicate that the application of pulse plating did not lead to any enhancement of the co-deposition rate of TiO_2_ nanoparticles as compared to DC deposits (Figure 2a). However, at lower peak current densities (J_p_) of 1 and 2 A/dm^2^ under pulse current (PC) conditions, the highest incorporation percentage of embedded TiO_2_ nanoparticles (2.56 ± 0.1 wt.%) was achieved when employing a low pulse frequency (0.1 Hz). Conversely, for J_p_ equal to 5 A/dm^2^, application of a high pulse frequency equal to 100 Hz facilitated the embedment of TiO_2_ nanoparticles into the alloy matrix. It is worth noting that the pulse frequency of ν = 1 Hz appeared to be the lower threshold for TiO_2_ incorporation, regardless of the applied peak current density, J_p_.

These results suggest that when applying a low pulse frequency (ν < 1 Hz) and a duty cycle of 50% (t_on_ = t_off_), the nanoparticles have more opportunities to be engulfed in the dual layer. During prolonged off-time (t_off_ > 0.5 s), the TiO_2_ nanoparticles that are loosely absorbed onto the cathode’s surface are excised, presenting increased potential to enter the dual layer and enhance the co-deposition rate [26].

At a pulse frequency of ν = 1 Hz, the duration of the imposed current (t_on_) was equal to 0.5 s, potentially resulting in a higher reduction rate of metal ions compared to the ions adsorbed onto the titania’s nanoparticles. This could lead to a reduction in the TiO_2_ content in the coating [21,27,28].

At higher current density values associated with a faster reduction rate of metal ions on the cathode’s surface, the application of higher pulse frequencies (v ≥ 10 Hz) resulted in an increased co-deposition percentage. When a higher pulse frequency was applied, the relaxation time (t_on_ = t_off_) was considerably shorter, typically less than 50 ms, allowing for an effective discharge of the electric double layer formed around the cathode. This facilitated better penetration of the ions adsorbed on the nanoparticles towards the cathode, resulting in higher deposition rates [26,29].

Moreover, it is important to consider that the applied overpotential is one of the predominant parameters influencing the reduction process. When the highest applied frequency (v ≥ 100 Hz) is used, it leads to a high instantaneous current spike induced at the beginning of the T_on_ period. This may be associated with the increased embedding rate of TiO_2_ nanoparticles in the alloy matrix observed in the pulse-electrodeposited composite coatings [30].

Furthermore, it is worth noting that the selection of applied pulse parameters has an impact on the composition of the deposits [31]. Figure 3b presents the Sn content (wt.%) in metal matrix as a function of pulse frequency at various pulse current density values.

In pulse plating, higher instantaneous current densities are achieved compared to direct current (DC) plating [32]. As a result, the metals tend to deposit at higher current density values. For the Sn–Ni electrodeposited coatings, previous studies indicate that during pulse electrodeposition, an increase in the pulse current density causes a small decrease in the tin content in the alloy matrix [19]. In the case of Sn–Ni/TiO_2_ pulse-plated deposits, regardless of the applied pulse current frequencies and pulse current density values, the reduction in Ni ions seems to be favored, resulting in lower Sn content in the matrix as compared to DC deposits. However, among the pulse-plated deposits, the application of J_p_ = 1 A/dm^2^ and a pulse frequency up to 1 Hz led to a reduction in Sn content compared to DC deposits. A further increase in pulse frequency resulted in a slight increase in the tin content (Figure 3b). This phenomenon can be explained by considering that the concentration gradient of tin near the cathode is higher under transient conditions (high frequencies) than during steady-state diffusion (DC or low frequencies). This higher concentration gradient allows for a greater deposition rate of Sn at shorter pulse periods. Similar observations have been reported previously for the pulse deposition of alloy coatings [31].

### 3.2. Surface Morphology and Structural Analysis of Sn–Ni/TiO_2_ Composite Coatings

**Figure 4:**
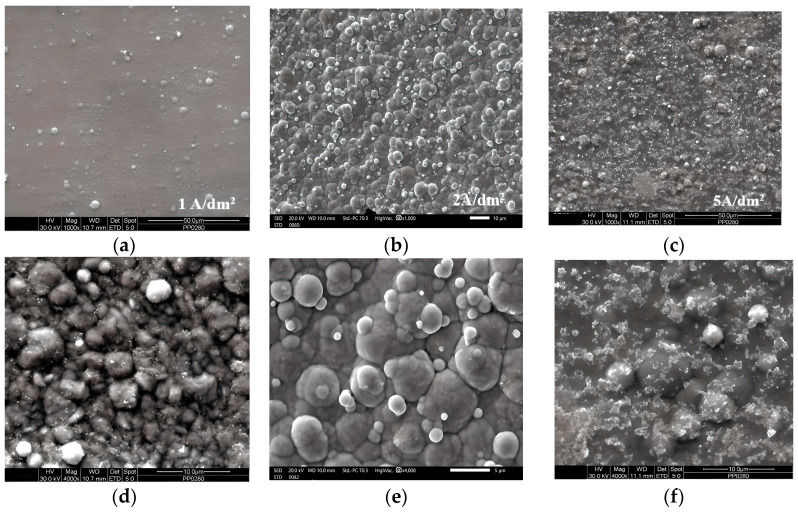
Effect of current density on the surface morphology of Sn–Ni/TiO_2_ composite coatings produced under DC conditions at (**a**,**d**) 1 A/dm^2^, (**b**,**e**) 2 A/dm^2^, and (**c**,**f**) 5 A/dm^2^.

Figure 4 presents the effect of the applied current density on the surface morphology of the Sn–Ni/TiO_2_ composite coatings produced under DC conditions.

The surface morphologies of the produced composite coatings differ significantly, depending on the current density and the amount of TiO_2_ nanoparticles incorporated into the metal matrix. The Sn–Ni/TiO_2_ composite coatings demonstrate a characteristic cauliflower-like structure [4,8,10,26]. Specifically, when the composite is produced under a low current density (J = 1 A/dm^2^), it is characterized by globular structures with well-defined grain boundaries (Figure 4a,d). As the current density increases to 2 A/dm^2^, there is an enhancement In the TiO_2_ concentration within the alloy matrix. Larger globular features become dominant in the surface of the deposit, and some nanoparticles form agglomerates (Figure 4b,e). These agglomerates are typically attributed to randomly dispersed TiO_2_ aggregates on the composite [33]. At the highest applied current density (5 A/dm^2^), where the TiO_2_ co-deposition percentage reaches one of its maximum values (2.5 wt.% TiO_2_), the surface morphology undergoes significant changes. The grain boundaries appear less distinct, and a “cloud” of flakes seems to protrude from the surface and the grains themselves (Figure 4c,f).

**Figure 5:**
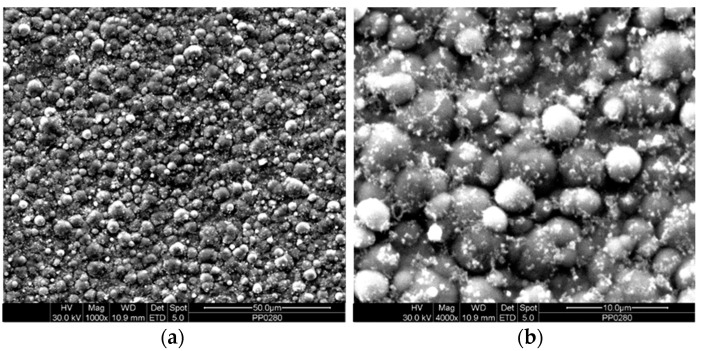
SEM micrographs of the Sn–Ni/TiO_2_ composite coating with the highest TiO_2_ co-deposition rate produced under PC conditions, d.c. 50%, ν = 0,1 Hz, J_p_ = 1 A/dm^2^, (**a**) magnification (×1000) and (**b**) magnification (×4000).

Regarding the surface morphology of the pulse-plated composite coatings, particular attention was paid to those produced under electrodeposition conditions that yielded the highest TiO_2_ incorporation rate in the Sn–Ni alloy matrix. Figure 5 demonstrates the morphological characteristics of the composite coating with the highest co-deposition rate (2.56 wt.% TiO_2_) obtained under pulse current conditions, specifically a pulse frequency of 0.1 Hz and a peak current density of 1 A/dm^2^. The surface of the coating appears to be covered with spherical nanoparticles ranging in size from 1 to 5 µm, and these nanoparticles are uniformly distributed across the entire surface of the coating.

**Figure 6:**
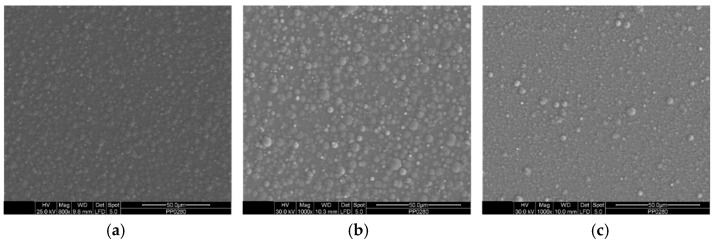
Effect of pulse frequency on the surface morphology of Sn–Ni/TiO_2_ composite coatings produced at, d.c. 50%, J_p_ = 1 A/dm^2^ at (**a**) ν = 1 Hz, (**b**) ν = 10 Hz, and (**c**) ν = 100 Hz.

Additionally, Figure 6 provides insights into how increasing the pulse frequency at a constant J_p_ = 1 A/dm^2^ affects the morphology of the composite coatings. The surface displays fewer globular features compared to the composite produced under pulse conditions (PC) at 0.1 Hz (Figure 5a). As the frequency increases, both the ‘on-time’ and ‘off-time’ become shorter, and the number of pulses at constant time increases. At the start of each pulse, new nuclei are created, and due to the short ‘on time’, these nuclei do not have sufficient time to grow. Consequently, fewer coarse points are prone to being formed on the surface of the coating [34].

The assessment of surface roughness of the composite coatings fabricated under DC conditions revealed a significant influence of the TiO_2_ incorporation ratio. The sample with the lowest TiO_2_ incorporation percentage (produced under J = 1 A/dm^2^) displayed the lowest roughness, with a Ra value of 0.16 ± 0.001 μm. Conversely, the sample with the highest TiO_2_ incorporation percentage (produced under J = 2 A/dm^2^) exhibited a roughness equal to 0.23 ± 0.02 μm (Ra). The application of J = 5 A/dm^2^ led to Ra = 0.21 ± 0.02 μm. These values are also consistent with the morphological characteristics of the SEM micrographs, as depicted in Figure 4.

Additionally, the effect of the application of pulse current on the surface roughness is presented in Table 3. The data show that composite coatings electrodeposited under different frequencies (0.1 to 100 Hz) exhibit higher roughness in comparison to the DC composite coating produced under the same applied current density.

**Figure 7:**
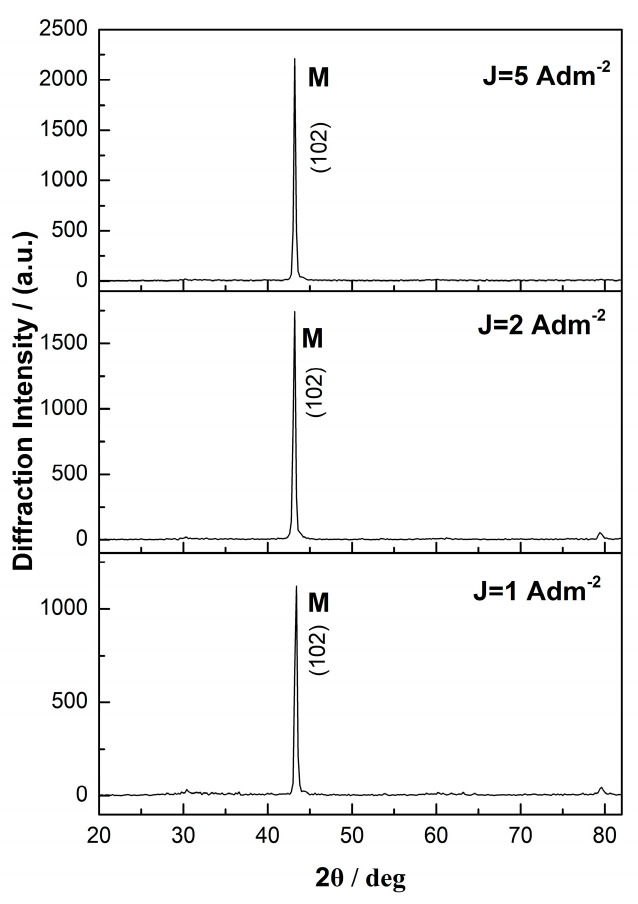
X−ray diffraction patterns of Sn–Ni/TiO_2_ composite coatings produced under DC conditions at J = 1, 2, and 5 A · dm^−2^.

**Figure 8:**
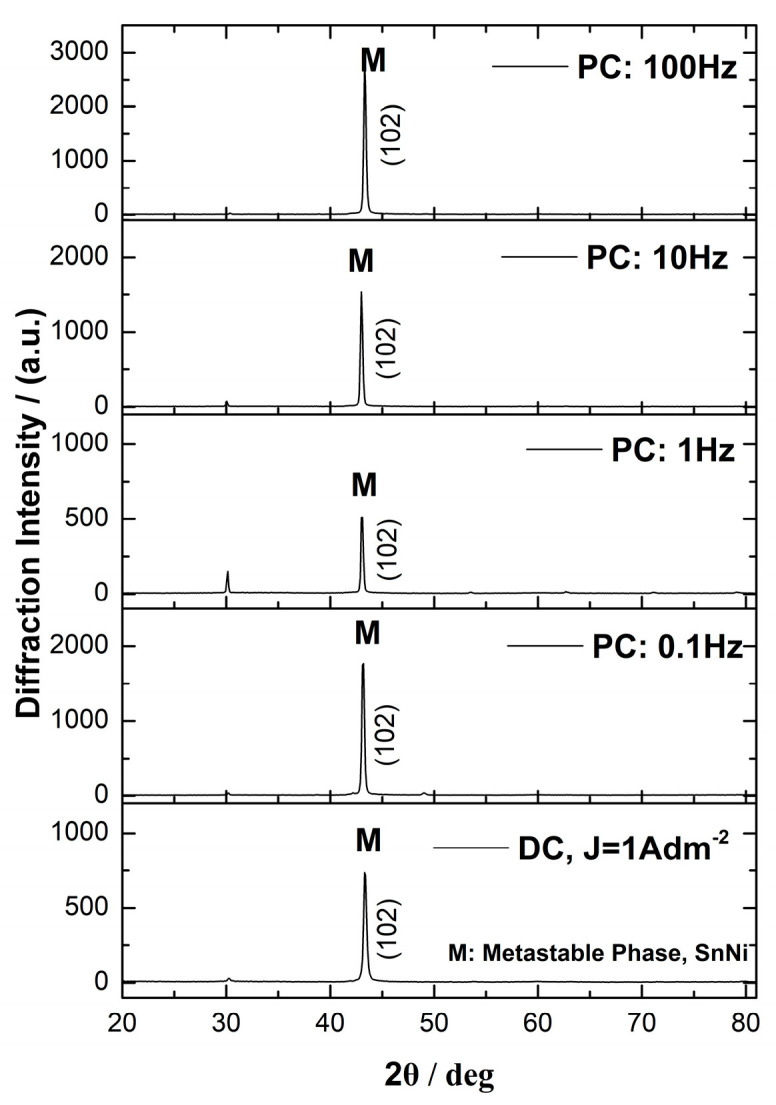
Effect of pulse frequency on X−ray diffraction patterns of Sn–Ni/TiO_2_ composite coatings produced under at J_p_ = 1 A · dm^−2^ d. c = 50% and ν = 0.1, 1, 10, and 100 Hz (DC provided for comparison).

The X-ray diffraction (XRD) patterns of the Sn–Ni/TiO_2_ composites produced under direct current (DC) and pulse current (PC) conditions are presented in Figure 7 and Figure 8, respectively.

In the X-ray diffraction analysis of the composite coatings, the main peak was detected at 2θ ≈ 43–43.34°, which is attributed to the (102) lattice plane of the metastable NiSn phase. In addition, a small peak at 2θ ≈ 30° is assigned to the (101) diffraction plane of the metastable SnNi phase [1,8,35]. The low-intensity peak at 2θ ≈ 80° is attributed to tin. Figure 7 illustrates that during direct current electrodeposition, as the applied current density increases, the intensity of the main metastable NiSn peak also increases. Overall, under DC conditions, the incorporation of the commercial TiO_2_ nanoparticles in the matrix does not significantly alter the crystalline structure, as the X-ray diffraction patterns closely resemble those of the pure electrodeposits (refer to Figure 3 in ref. [8]).

In Figure 8, under pulse current condition J_p_ = 1 A/dm^2^, composite coatings produced under ν = 0.1, 1, and 10 Hz exhibit a slight shift of the main peak towards lower angles. This shift is associated with the increase in the lattice constant (interplanar spacing) presented in Table 4.

It is well known that at higher pulse frequencies, the kinetics of the electrodeposition process may be altered. Faster pulses can influence the mass transport of metal ions to the electrode surface and their incorporation into the crystal lattice. These changes in mass transport can affect the arrangement of atoms within the crystal lattice, potentially leading to increased interplanar spacing [36]. It is worth noting that the composite coatings produced under pulse frequencies of ν = 0.1, 1, and 10 Hz possessed an Sn content of 65 wt.%, in contrast to the coatings produced under ν = 100 Hz, where the Sn content was 67 wt.%, similar to DC-plated coatings (Figure 3b). In a related study by Rooksby in 1950 [36], it was mentioned that during the electrodeposition process, additional tin could be incorporated into the alloy, and the lattice sites that are typically unoccupied may become largely filled. This incorporation of additional tin into the lattice structure could be accompanied by lattice strains [37]. The crystalline experimental findings for the pulse-plated composites at ν = 0.1, 1, and 10 Hz could support the aforementioned interpretation of the electrocrystallization process of Sn–Ni composite coatings.

The average grain size was determined using Scherrer’s formula, based on the main diffraction peak at 2θ ≈ 43.6°, which corresponds to the metastable NiSn phase. The X-ray diffraction analysis revealed that all composite coatings exhibited a nano-crystalline structure. The estimation of grain size from the XRD analysis indicated that the average crystalline size of the direct current (DC) composite coatings was consistent, approximately 36–38 nm, and did not significantly change with increasing applied current density. The application of pulse electrodeposition at J_p_ = 1 A/dm^2^ led to a slight decrease in the crystalline size of the composite coatings (Table 4). This observation aligns with previous studies, where the pulse electrodeposition technique was shown to be an effective method for producing finer-grained Ni-based composite electrodeposits [19,21].

Interestingly, the incorporation of TiO_2_ nanoparticles resulted in a notable increase in the crystallite size compared to pure Sn–Ni coatings produced using the same electrolytic bath. The pure Sn–Ni coating exhibited an average grain size of approximately 22 nm under these conditions [8]. This difference is likely attributed to the anchoring of TiO_2_ nanoparticles in the inter-crystallite sites, which appears to influence the nucleation and growth processes of the Sn–Ni deposit. A similar effect was noted in the case of Ni–Co/TiO_2_ composites [33].

### 3.3. Microhardness

**Figure 9:**
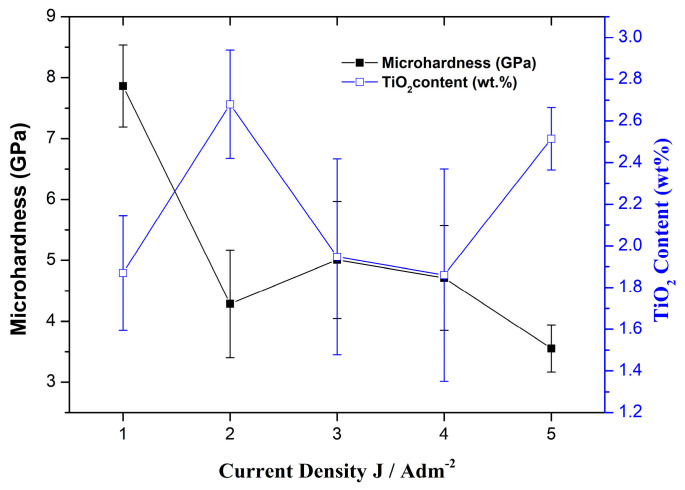
Vickers microhardness vs. TiO_2_ content in deposit for Sn–Ni/TiO_2_ composite coatings produced under DC conditions as a function of current density J (A/dm^2^).

The microhardness of the Sn–Ni/TiO_2_ composite coatings as a function of current density under direct current electrodeposition is presented in Figure 9. The highest microhardness value was achieved at low current density (J = 1 A/dm^2^) (7.86 ± 0.67 GPa), whereas the lowest microhardness among the coatings produced under direct current (DC) conditions was obtained at 5 A/dm^2^ (3.55 ± 0.39 GPa). However, at current densities equal to 2 A/dm^2^ and 5 A/dm^2^, where the TiO_2_ incorporation percentage reached its maximum, the microhardness values were significantly lower. As a result, increased levels of embedded TiO_2_ nanoparticles within the alloy matrix seem to be associated with decreased microhardness values.

In this type of composite coatings, it seems that the increased amounts of co-deposited TiO_2_ nanoparticles are not uniformly distributed within the alloy matrix, thus allowing a dispersion hardening effect that consequently improves the coatings’ hardness. Hence, the incorporation of TiO_2_ in the matrix could lead to increased agglomeration of nanoparticles, which could be associated with a reduction in coating hardness [38]. On the other hand, it is apparent that the application of low current density (1 A/dm^2^), which is associated with slower deposition rates, results in a more uniform distribution of particles in the metallic matrix. This uniform distribution contributed to a significant increase in hardness, potentially through a dispersion hardening mechanism [29].

**Figure 10:**
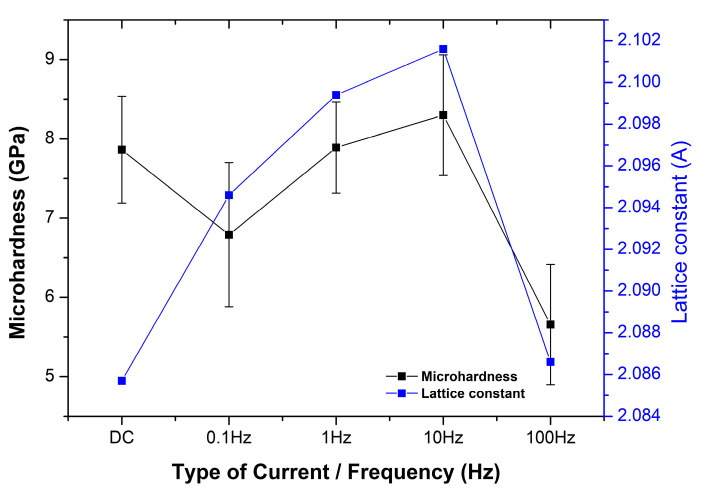
The relationship of Vickers microhardness and lattice constant of Sn–Ni/TiO_2_ composite coatings as a function of applied pulse frequency (J_p_ = 1 A/dm^2^).

As mentioned in Section 3.1, the application of pulse electrodeposition (J_p_ = 1 A/dm^2^) enhanced the incorporation of TiO_2_ nanoparticles compared to DC conditions. The impact of pulse frequency on the microhardness of these composite coatings was examined, as presented in Figure 10.

Under pulse current conditions, the highest microhardness value was achieved at an applied frequency equal to 10 Hz (8.3 ± 0.76 GPa), while the lowest occurred at 100 Hz (5.66 ± 0.75 GPa). Figure 10 indicates a proportional relationship between pulse frequency, lattice constant, and microhardness. Increasing the pulse frequency from ν = 0.1 Hz to 10 Hz resulted in both an increase in the lattice constant and in the microhardness. The coating with the highest microhardness value also exhibited the highest lattice constant (ν = 10 Hz). It should be noted that the coating with the highest TiO_2_ incorporation percentage among those produced under pulse conditions (ν = 0.1 Hz) did not exhibit enhanced microhardness. This finding reveals that in this kind of composite coating, the hardening mechanism is more influenced by the structural characteristics of the alloy matrix rather than the quantity of embedded particles and/or their corresponding uniform dispersion in the alloy matrix. In addition, support for the previous interpretation could be achieved through the experimental observation that a further increase in the pulse frequency led to a decrease in the Sn content accompanied by an increment in the lattice constant, resulting in a significant increase in the microhardness. The highest applied frequency of 100 Hz is accompanied by a decrease in the lattice constant, resulting in a significant reduction in the microhardness. The application of higher frequency decreases the relaxation time during t_off_, and it is possible that stresses are induced in the crystal lattice, provoking a decrease in the microhardness of the coating.

In previous studies, it has been reported that enhancing coatings’ properties utilizing PC plating is not always associated with the influence of pulse parameters on the particle co-deposition processes [20]. The experimental data of this study reveal that it is of high significance to consider the crystal characteristics of the coatings (structure, strain, stresses, average crystalline size), in order to determine if there is a direct correlation between the co-deposition rate of nanoparticles in the matrix and microhardness.

### 3.4. Wear Behavior of Sn–Ni/TiO_2_ Composite Coatings

**Figure 11:**
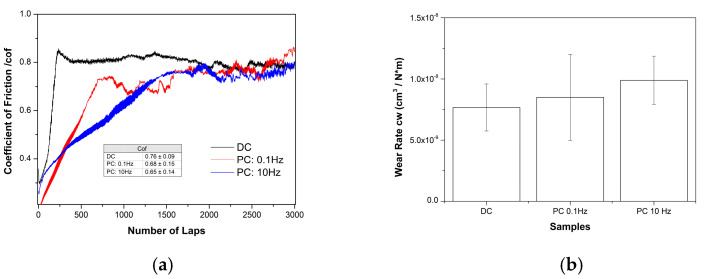
(**a**) Evolution of friction coefficient and (**b**) volumetric wear rate of Sn–Ni/TiO_2_ composite coatings sliding against a 100Cr6 ball (d = 6 mm).

In general, the incorporation of nanoparticles into the matrix of composites aims to enhance the tribological properties of nickel-based alloy coatings [6]. In this study, the tribological properties were assessed for the Sn–Ni/TiO_2_ composite coatings that exhibited the highest microhardness values under DC and PC conditions (DC: J = 1 A/dm^2^, PC: ν = 10 Hz, J_p_ = 1 A/dm^2^), as well as for the composite with the highest TiO_2_ incorporation percentage (PC: ν = 0.1 Hz, J_p_ = 1 A/dm^2^). The evolution of the dry sliding friction coefficient (cof = μ) was recorded as a function of the number of laps (Figure 11a).

The mean values of the recorded coefficient of friction (μ) during the entire test period show that the DC composite coating exhibits a slightly higher μ value (i.e., μ = 0.76) compared to the PC composites (μ = 0.65–0.68). These tested composite coatings showed several differences in their TiO_2_ incorporation percentage, Sn content, roughness, and microhardness, which may have influenced their coefficient of friction.

**Figure 12:**
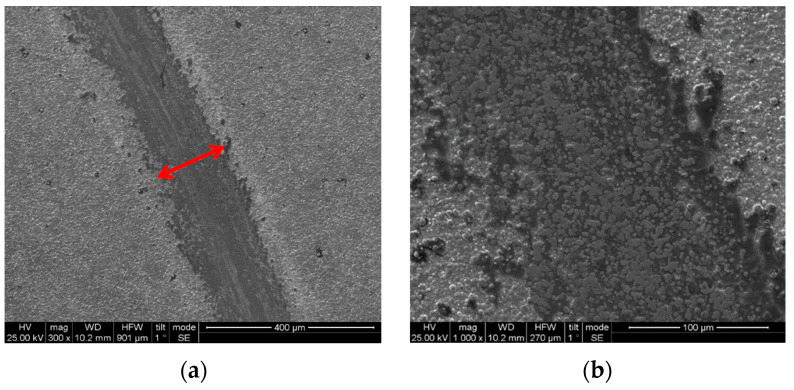

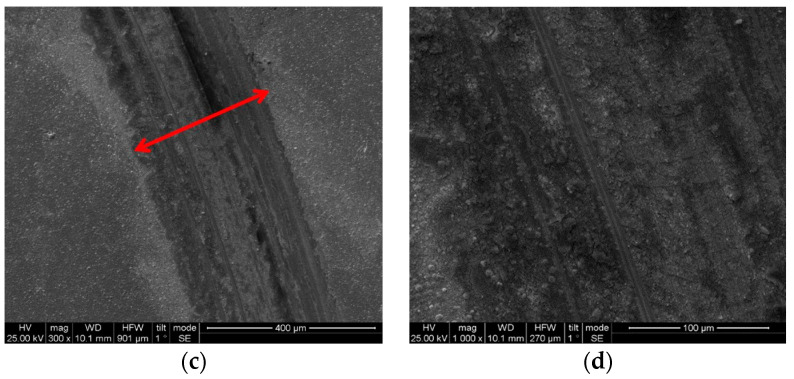
Comparison of the SEM surface micrographs after tribological test of (**a**) the wear track (magnification ×300), (**b**) the worn surface (magnification ×1000) of the composite produced under DC conditions with J = 1 A/dm^2^, (**c**) wear track (magnification ×300), and (**d**) worn surface (magnification ×1000) of the composite produced under PC, with J_p_ =1 A/dm^2^, d.c.: 50%, ν = 0.1 Hz (red arrows depict the width of the wear track).

**Figure 13:**
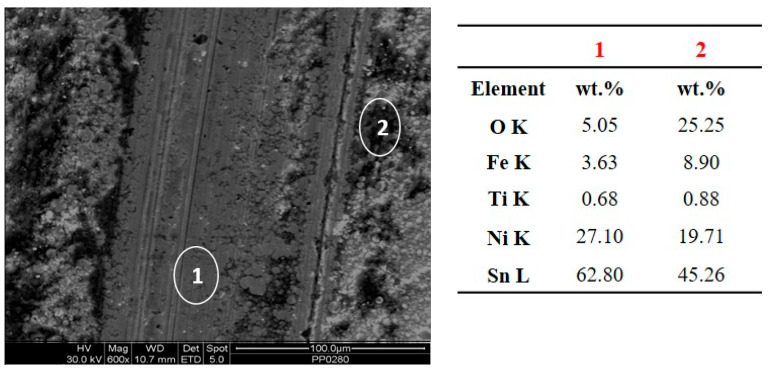
SEM micrographs inside the wear track and EDS analysis at spots 1 and 2 of the Sn–Ni/TiO_2_ composite coating produced under DC, J = 1 A/dm^2^.

**Figure 14:**
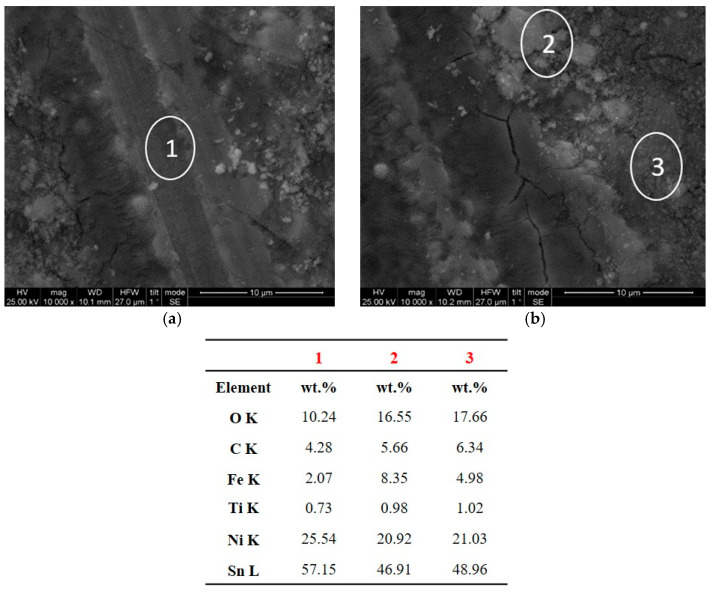
(**a**,**b**) SEM micrographs within the wear track of the Sn–Ni/TiO_2_ composite coating produced under PC, J_p_ = 1 A/dm^2^, d.c.: 50%, ν = 0.1 Hz, and corresponding EDS analysis at spots 1, 2, and 3.

The evaluation of volumetric wear rate c_w_ (Figure 11b) demonstrated that the PC composite coating produced at ν = 10 Hz exhibits the highest microhardness and a higher surface roughness value, and the lowest wear resistance among the tested composites. It appears that increasing the amount of incorporated TiO_2_ in the coating gradually decreases the wear resistance. This may be due to the fact that the incorporated TiO_2_ nanoparticles exhibit a load-carrying effect that simultaneously reduces direct contact between the surface of the Sn–Ni matrix and the opponent ball [6]. The best wear resistance was recorded for the DC plated composite, which exhibited the highest coefficient of friction (μ) and the lowest surface roughness (Table 3). Beyond an optimum concentration, increasing the TiO_2_ concentration can lead to an increase in the wear rate due to the formation of a composite coating with a porous structure [6]. Additionally, the tribological characteristics of the composites were examined using scanning electron microscopy, and the morphology of typical wear tracks is presented in Figure 12, Figure 13 and Figure 14.

**Figure 15:**
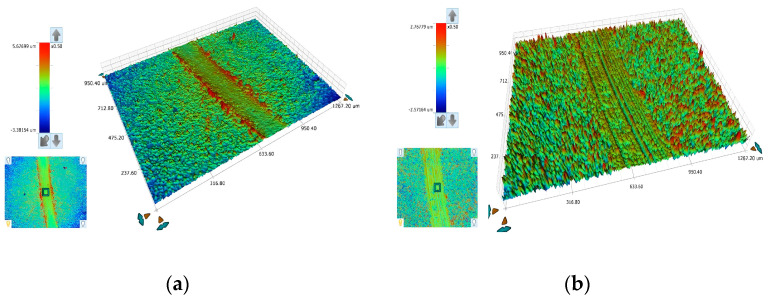
3D and 2D plots of the wear tracks of Sn–Ni/TiO_2_ composite coating produced under (**a**) DC, J = 1 A/dm^2^ and (**b**) PC, J_p_ = 1 A/dm^2^, d.c.: 50%, ν = 0.1 Hz.

The width of the wear track of the DC composite coating was smaller compared to that of the PC composite coating (Figure 12). In the case of the DC composite coating, a debris layer formed on both sides of the wear track (Figure 13 and Figure 15a). The EDS analysis near the debris layer (Figure 13, Spectrum 2) showed high oxygen content that verifies the oxide nature of the debris. Additionally, the detection of iron (Fe) indicated the material transfer from the opposing 100Cr6 ball during the sliding tests.

In the case of the composite coating produced under PC conditions at ν = 0.1 Hz, the wear track presented scratches (Figure 12c,d and Figure 15b) parallel to the direction of motion, revealing the existence of abrasive wear. Additionally, the wear track exhibits brittle fracture cracks vertical to the direction of motion, which are typical of brittle fracture wear (Figure 14a) [8,39]. On both sides of the worn surface, uniformly distributed asperities were overlaid and wear debris adhered to the wear track surface, resulting in the formation of numerous micro-cracks, as detailed in Figure 14. Moreover, an EDS analysis conducted on the edges of the wear track unveiled a significant transfer of material from the opponent ball to the composite (Figure 14, spots 1, 2, 3), suggesting an increased resistance to dry sliding. The EDS analysis within the wear track of the composite coating (Figure 14, spots 1, 2, 3), indicated a notable oxygen content, signifying the generation of oxide wear debris, a characteristic feature of oxidative wear processes [40].

In summary, the wear behavior of the composite coatings under dry sliding conditions against a steel ball showed the occurrence of abrasion, adhesion, and oxidation phenomena. The best tribological performance was observed for the composite coating produced under DC conditions at a current density of 1 A/dm^2^.

### 3.5. Photocatalytic Performance of Composite Coatings

**Figure 16:**
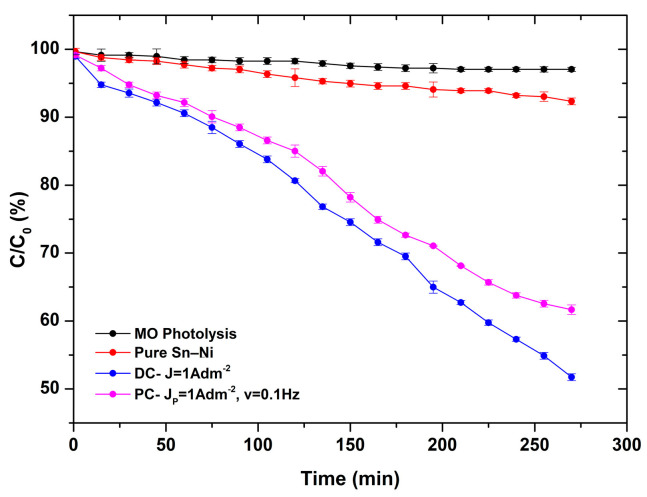
Degradation curves of MO as a function of time under UV light irradiation for pure and composite Sn–Ni/TiO_2_ coatings. The photolysis of MO is also included. Lines present polynomial regression fitting curves.

The photocatalytic performance of composite coatings with immobilized Evonik P-25 TiO_2_ nanoparticles within Sn–Ni alloy matrix was assessed by studying the degradation of methyl orange under UV irradiation. Figure 16 demonstrates the degradation curves of dye solutions at various time intervals for selected composite coatings. The dye adsorption of the reference sample (pure Sn–Ni coating) is also included, as well as the photolysis of MO exhibiting a degradation percentage < 10%. The composite coatings tested were the composite coatings produced under direct current conditions, DC at J = 1 A/dm^2^ (1.86 wt.% TiO_2_), and the pulse-plated composite coating (PC) exhibiting the highest incorporation of TiO_2_ nanoparticles (2.56 wt.% TiO_2_) produced at J_p_ = 1 A/dm^2^, d.c. 50%, and ν = 0.1 Hz.

**Figure 17:**
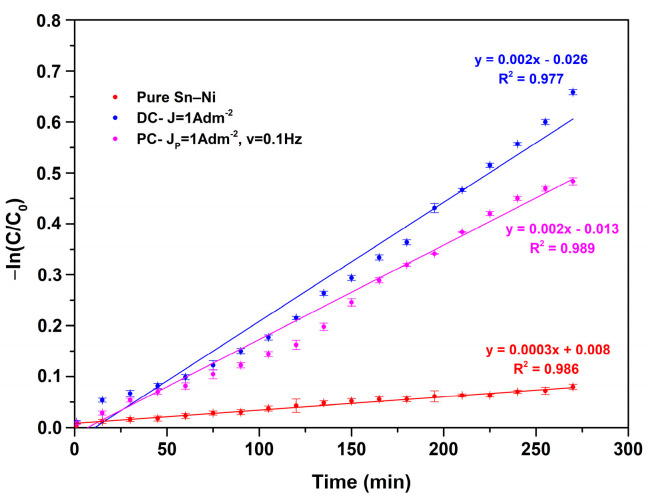
Photocatalytic kinetics under UV light irradiation for methyl orange for pure and composite Sn–Ni/TiO_2_ coatings.

As expected, the pure sample (Figure 16) did not show any significant photocatalytic activity, and the obtained MO degradation values were rather close to those corresponding to photolysis. Nevertheless, the slight absorbance noted could be associated either with the activity of the oxide layer formed on the upper surface of the alloy coating, or with specific surface characteristics such as the roughness of the pure coatings. The composite coating produced under direct current conditions exhibited better photocatalytic activity, as proven by the MO degradation percentage of approximately 50% at 260 min of irradiation, compared to the PC sample where almost 40% MO degradation was achieved (Figure 16). The photocatalytic kinetics for the pure and the composite coatings are presented in Figure 17.

The experimental data exhibit a good fit with the kinetics model, with a coefficient of determination (R^2^) greater than 90%, indicating satisfactory adaptation of the model to the data [41]. The kinetics model observed in this case is characterized as pseudo-first-order. According to the Langmuir–Hinshelwood model, this is the type of kinetics that azo dyes typically follow during photodegradation processes [42].

Regarding the photocatalytic activity of immobilized TiO_2_ catalyst in Ni metal matrix composite coatings, it has been presented that the higher TiO_2_ content in the deposit is associated with a higher decomposition rate of the pollutant [9]. However, this contrasts with our current findings, as the sample with the lowest TiO_2_ content among tested samples had the best photocatalytic performance. It is known that the catalytic efficiency often changes based on the hosting metal [43]. Since the structural characteristics (Sn–Ni content, grain size, crystallographic orientation) of the matrix of both composites are similar, it seems that the TiO_2_ concentration and distribution of the nanoparticles in the metal matrix plays a crucial role in their photocatalytic performance. It is possible that the higher TiO_2_ content leads to the formation of TiO_2_ aggregates, which may prevent the active centers from receiving light irradiation, consequently hampering the photocatalytic activity of TiO_2_ [44,45]. However, it is important to note that compared to Ni/TiO_2_ composites [9], the photocatalytic performance of Sn–Ni/TiO_2_ with immobilized Evonik P25 TiO_2_ nanoparticles shows enhanced photocatalytic performance, rendering this type of coating appealing for applications with self-cleaning properties.

### 3.6. Corrosion Resistance

In order to study the corrosion behavior and passivation properties of Sn–Ni/TiO_2_ composite coatings, a test solution of 3.5% NaCl at pH = 6 was used at room temperature. The experimental procedure for all corrosion assessments followed a standardized sequence. Initially, the corrosion potential (also known as open circuit potential—OCP) was monitored until it reached a stable value. Subsequently, linear polarization curves were recorded, and the polarization resistance (R_p_) slope was determined through linear regression analysis. R_p_ serves as an indicator of the effectiveness of the protective passivation layer on the alloy surface. The R_p_ values are presented in Table 5.

**Figure 18:**
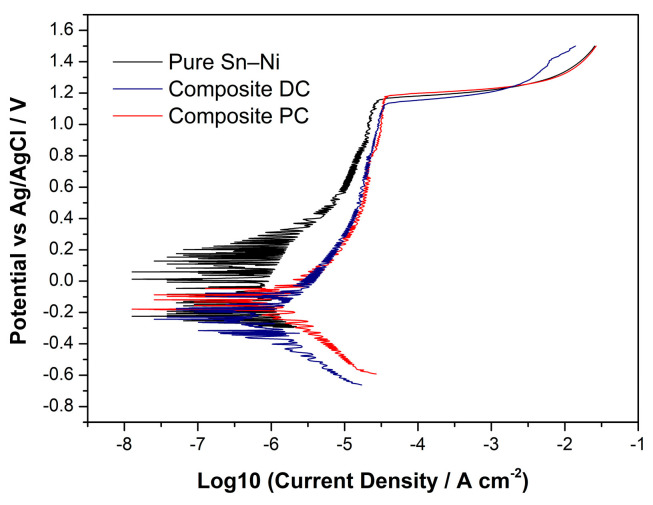
Dynamic polarization curves for pure Sn–Ni (black), DC composite Sn–Ni/TiO_2_ (red), and PC at ν = 0.1 Hz composite Sn–Ni/TiO_2_ (blue) in 3.5% NaCl at 293 K, obtained using a potential scan rate of 1 mV/s.

The potentiodynamic polarization curves were employed to examine the passivation behavior of Sn–Ni/TiO_2_ deposits at room temperature, and the results are presented in Figure 18. For the sake of comparison, we conducted identical experiments on a pure Sn–Ni deposit with equivalent thickness. 

As presented in Table 5, the corrosion potential of Sn–Ni/TiO_2_ composite coatings is more negative compared to pure Sn–Ni coating, indicating a less noble behavior for the composite coatings. Between the two composites, the DC deposit demonstrates a more negative corrosion potential, indicating a higher tendency for corrosion; this is probably due to the higher Sn content recorded in the metal matrix. However, the corrosion current that is directly proportional to the corrosion rate of the coatings could not be estimated through extrapolation from the Tafel slope intercepts, since the actual corroding area remains unknown due to the ongoing passivation phenomena occurring in the anodic region of the polarization curves (Figure 18). Regarding the passivation of Sn–Ni/TiO_2_ coatings, previous research studies [39,46,47] have shown that the electrodeposited Sn–Ni coatings consisting of the single-phase Sn–Ni compound can develop passivity across a broad range of corrosion environments and pH levels, where the parent metals suffer rapid attack [46]. In this context, the potential range situated between the corrosion potential (E_corr_(i = 0)) and the breakdown potential E_bd_ (approximately +1100 mV vs. Ag/AgCl) represents the passivation zone, where corrosion is minimal or even negligible. Beyond the E_bd_ potential, there is a sharp increase in the anodic current for all types of coatings. This trend suggests the breakdown of the passive film, indicating the onset of pitting corrosion and further anodic dissolution of the coating. Additionally, the fluctuation in current density observed in Figure 18, especially for the pure coating prior to sustained breakdown, may be attributed to metastable pitting. These current spikes result from corrosion pits that ultimately re-passivate, unlike stable pitting where the passive film cannot reform over the pits [48,49].

It is worth noting that the anodic polarization behavior of the two individual metals, when tested under similar conditions, differs significantly from that of the Sn–Ni alloy. Notably for nickel, the anodic polarization curve in a 3% NaCl solution [50] indicates that no passivation zone is observed above the corrosion potential at approximately −0.250 mV vs. SHE (−0.50 mV vs. Ag/AgCl). Anodic dissolution takes place with a continuous increase in corrosion current density. In contrast, tin exhibits a more negative corrosion potential at approximately −0.450 mV vs. SHE (−0.250 mV vs. Ag/AgCl). Tin shows extended passivation within the range from −0.400 mV up to 0 mV vs. SHE, while above 0 mV vs. SHE, there is an increase in corrosion current density, suggesting the anodic dissolution of the metal. Concerning corrosion and passivation behavior, the tin–nickel coating exhibits distinct characteristics compared to both tin and nickel. The extension of the passive zone reduces the alloy’s risk of rapid corrosion, through either polarization or galvanic effects resulting from the presence of another alloy.

**Figure 19:**
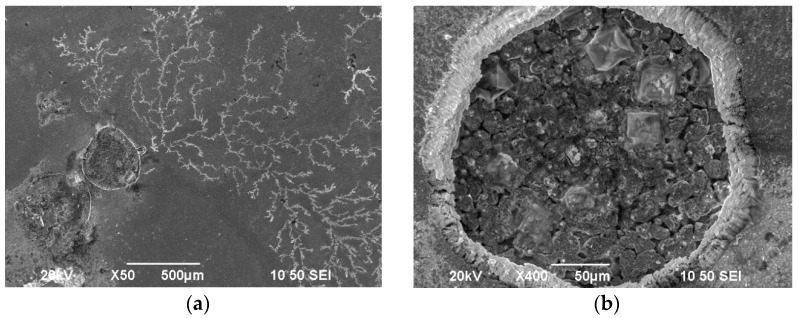
SEM surface micrographs, at (**a**) magnification (×50) and (**b**) magnification (×400), of the Sn–Ni/TiO_2_ composite coating produced under DC conditions at J = 1 A/dm^2^ after potensiodynamic polarization in 3.5% NaCl.

After potentiodynamic polarization, the surface morphology of the samples was examined using SEM (Figure 19a,b). The surface features are indicative of pitting corrosion, characterized by pronounced localized craters with diameters reaching approximately 500 μm (Figure 19a). These craters likely begin forming when a chemical breakdown process (e.g., point defects or scratches) exposes a specific location on the metal surface to ions, such as chloride ions. The craters continue to grow if rapid re-passivation fails to halt the accumulation of high concentrations of metal ions produced by dissolution at the point of initiation.

**Figure 20:**
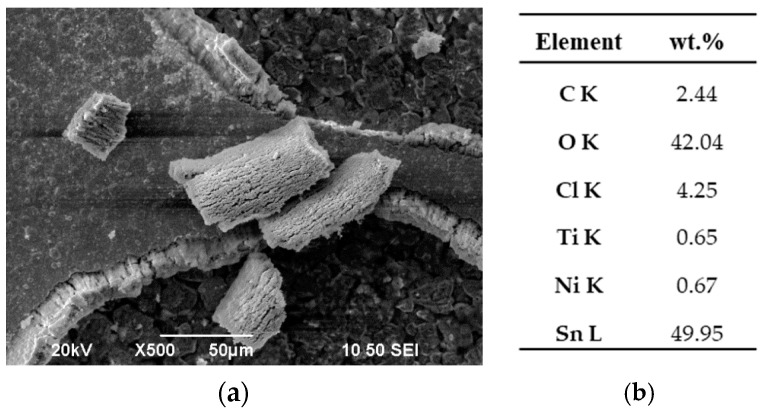
(**a**) SEM surface micrograph of the Sn–Ni/TiO_2_ composite coating produced under DC conditions at J = 1 A/dm^2^ after potensiodynamic polarization in 3.5% NaCl, and (**b**) elemental analysis of white area broken piece of the coating.

In Figure 20, the surface micrograph of broken pieces of the surface material is provided, accompanied by the corresponding elemental analysis. The results indicate that the white areas, representing the broken pieces, primarily consist of tin and oxygen. Previous corrosion studies on Sn–Ni coatings have reported the formation of a passive layer at very low potentials, which has been identified to contain a few monolayers of compounds, such as SnO_2_, Sn-OH, and Ni-OH [16,46,51]. Importantly, this passive layer remains unaffected by the presence of chloride ions over a wide range of applied anodic potentials [16,39,46]. Based on the above data, it is plausible that the Sn-rich oxides formed during the passivation of the coating may have broken down after prolonged exposure to current flow from the surface.

Overall, composite coatings have a similar corrosion trend to pure coatings (passivity and subsequent pitting corrosion). However, the incorporation of TiO_2_ nanoparticles does not have a positive effect on the corrosion resistance of the composite coatings. Since TiO_2_ forms spherical agglomerates distributed in the surface of the coatings (see Figure 4a, micrograph i and Figure 5), it seems that regardless of the chemical inertness of the nanoparticles, the agglomerated coatings may act as sites of favored corrosion due to the non-uniform distribution within the metal matrix [52].

## 4. Conclusions

Direct and pulse electrodeposition techniques were applied to incorporate TiO_2_ nanoparticles into an Sn–Ni matrix, resulting in the production of Ni–Sn/TiO_2_ composite coatings with enhanced mechanical, wear resistance, photocatalytic, and anticorrosion properties. The study focused on two main aspects by varying the current density (DC and PC plating) and pulse frequency (for PC deposition): (i) the co-deposition rate of titania nanoparticles, and (ii) the composition of the alloy matrix within the composite coatings.

In terms of the TiO_2_ nanoparticle co-deposition rate, it was observed that the use of pulse current, compared to direct current electrodeposition, increases the incorporation percentage of TiO_2_ nanoparticles, particularly when a low peak current density (J_p_ = 1 A/dm^2^) is applied. The highest incorporation of TiO_2_ nanoparticles (2.56 wt.% TiO_2_) was achieved at J_p_ = 1 A/dm^2^, at 50%, and a pulse frequency (ν) of 0.1 Hz. Additionally, direct current electrodeposition revealed that the current density’s impact is more significant in determining the composition of the alloy matrix than its influence on the incorporation rate of TiO_2_. Moreover, during pulse electrodeposition, regardless of the applied pulse current frequencies and pulse current density values, the reduction in Ni ions appears to be favored, resulting in lower Sn content in the matrix compared to DC deposits.

The surface morphology of these composites is characterized by the cauliflower-like structure. The morphological characteristics of the composites varied depending on the applied current density, as well as on the amount of TiO_2_ nanoparticles incorporated into the metal matrix, with the surface roughness showing a significant influence from the TiO_2_ incorporation rate. Moreover, X-ray diffraction analysis showed that all of the composites are nano-crystalline, while the application of pulse current provoked alterations in the crystal lattice of the composite coatings.

In terms of mechanical properties, the microhardness evaluation demonstrated that for the DC deposits, higher levels of embedded TiO_2_ nanoparticles within the alloy matrix were associated with decreased microhardness values, while the application of pulse frequency led to a positive impact on the microhardness of Ni–Sn/TiO_2_ composite coatings. The wear behavior of composite coatings under dry sliding conditions against a steel ball showed that abrasion, adhesion, and oxidation phenomena dominated the tribological behavior of the composites.

For the photocatalytic activity under UV irradiation, it was concluded that the higher TiO_2_ content in the deposit is not associated with better photocatalytic performance. It is important to note that compared to Ni/TiO_2_ composites [9], the photocatalytic performance of Sn–Ni/TiO_2_ with immobilized Evonik P25 TiO_2_ nanoparticles shows enhanced photocatalytic performance, indicating that the distribution of the nanoparticles in the metal matrix is a crucial factor that influences the photo-induced catalytic process.

Regarding the corrosion resistance of these coatings in 3.5% NaCl, it was proven that the embedment of TiO_2_ nanoparticles into the tin–nickel matrix resulted in a reduction in the corrosion potential, indicating a less noble behavior for the composite coatings. Nevertheless, the composite coatings exhibited passivation, even at elevated anodic potentials.

Overall, in this study it is apparent that the application of direct current compared to pulse current electrodeposition favors the synthesis of Sn–Ni/TiO_2_ composites with enhanced mechanical, wear, photocatalytic, and anti-corrosion properties. The composite produced under DC electrodeposition demonstrated the best photocatalytic performance, preserved high microhardness values, and the lowest wear rate among the tested composites, and exhibited passivation even at elevated anodic potentials, rendering this type of coating appealing for applications with self-cleaning properties.

It is worth mentioning that the immobilization of the particles by electrodeposition on a metallic surface demonstrates advantages compared to related applications using dispersed powder catalysts, since it is mandatory to recycle and re-use the materials. Moreover, with this type of coating, the particles are stable within the matrix and simultaneously present enhanced mechanical, wear, and anticorrosion properties compared to films based on pure titania like spray-coatings, dip-coatings, etc., which suffer from mechanical instability issues (cracking and pores, poor adhesion on substrate, etc.) [53].

## Figures and Tables

**Table 1:** Electrodeposition parameters for the synthesis of Sn–Ni/TiO_2_ composite coatings.

Electrodeposition Parameters	Experimental Conditions
T:	70 ± 1 (°C)
pH:	4.2–4.4
Agitation:	Magnetic stirring 250 rpm
Rotation disc electrode:	600 rpm
Current density (J):	1 to 5 Adm^−2^
Duty Cycle (d.c) = T_on_/(T_on_ + T_off_):	50%
Frequency of pulses (ν):	0.1, 1, 10, 100 Hz
Pulse on/off time (s):	5, 0.5, 0.05, 0.005 s
Substrate:	Brass disc (diameter 25 mm)

**Table 2:** Experimental conditions for the Sn–Ni/TiO_2_ composite coatings’ tribological study.

Tribological Parameters	Experimental Conditions
Load	2Ν
Ball	100 Cr6 (d = 6 mm)
Sliding cycles	3000 Laps
Ball’s linear velocity	0.1 m/s
Ambient conditions	T = 25 °C, Humidity: 42–50%

**Table 3:** Effect of pulse frequency on the surface roughness of Sn–Ni/TiO_2_ composite coatings produced at applied current density J_p_ = 1 A/dm^2^.

Electrodeposition Conditions	Roughness (μm)
DC	0.16 ± 0.001
PC—0.1 Hz	0.21 ± 0.05
PC—1 Hz	0.25 ± 0.04
PC—10 Hz	0.29 ± 0.03
PC—100 Hz	0.23± 0.01

**Table 4:** Effect of pulse frequency on the lattice constant and average grain size of Sn–Ni/TiO_2_ composite coatings produced at an applied peak current density of J_p_ = 1 A/dm^2^.

Electrodeposition Conditions	Lattice Constant (Å)	Average Grain Size (nm)
DC	2.0857	36
PC—0.1 Hz	2.0946	32
PC—1 Hz	2.0994	34
PC—10 Hz	2.1016	32
PC—100 Hz	2.0866	32

**Table 5:** Corrosion parameters of Sn–Ni and Sn–Ni/TiO_2_ composite coatings in a 3.5% NaCl solution, as determined from the results of potentiodynamic measurements.

Sample	OCP (V)	J_pass_ (A cm^−2^)	R_p_ (Ohm/cm^2^)
Sn–Ni (Pure)	−0.037	2.84 × 10^−5^	0.56 × 10^6^
Sn–Ni/TiO_2_—DC	−0.135	3.48 × 10^−5^	0.1 × 10^6^
Sn–Ni/TiO_2_—PC 0.1 Hz	−0.08	3.43 × 10^−5^	0.38 × 10^5^

## Data Availability

Data are contained within the article.
